# *TRPM1* Mutations are the Most Common Cause of Autosomal Recessive Congenital Stationary Night Blindness (CSNB) in the Palestinian and Israeli Populations

**DOI:** 10.1038/s41598-019-46811-7

**Published:** 2019-08-19

**Authors:** Alaa AlTalbishi, Lina Zelinger, Christina Zeitz, Karen Hendler, Prasanthi Namburi, Isabelle Audo, Ruth Sheffer, Claudia Yahalom, Samer Khateb, Eyal Banin, Dror Sharon

**Affiliations:** 1 Department of Ophthalmology, Hadassah Medical Center, Faculty of Medicine, The Hebrew University of Jerusalem, Jerusalem, 91120 Israel; 2 St. John of Jerusalem Eye Hospital Group, East Jerusalem, 91198 Palestine; 3 Present Address: Neurobiology Neurodegeneration & Repair Laboratory, National Eye Institute, NIH, Bathesda, MD 20892, United States; 4 Sorbonne Université, INSERM, CNRS, Institut de la Vision, Paris, France; 5 CHNO des Quinze-Vingts, DHU Sight Restore, INSERM-DGOS CIC 1423, Paris, France; 6 Institute of Ophthalmology, University College of London, London, UK; 7 Department of Genetic and Metabolic Diseases, Hadassah-Hebrew University Medical Center, 91120 Jerusalem, Israel

**Keywords:** Molecular biology, Molecular biology, Retinal diseases, Retinal diseases, Genetics research

## Abstract

Precise genetic and phenotypic characterization of congenital stationary night blindness (CSNB) patients is needed for future therapeutic interventions. The aim of this study was to estimate the prevalence of CSNB in our populations and to study clinical and genetic aspects of the autosomal recessive (AR) form of CSNB. This is a retrospective cohort study of Palestinian and Israeli CSNB patients harboring mutations in *TRPM1* underwent comprehensive ocular examination. Genetic analysis was performed using homozygosity mapping and sequencing. 161 patients (from 76 families) were recruited for this study, leading to a prevalence of 1:6210 in the vicinity of Jerusalem, much higher than the worldwide prevalence. 61% of the families were consanguineous with AR inheritance pattern. Biallelic pathogenic *TRPM1* mutations were identified in 36 families (72 patients). Two founder mutations explain the vast majority of cases: a nonsense mutation c.880A>T (p.Lys294*) identified in 22 Palestinian families and a large genomic deletion (36,445 bp) encompassing exons 2–7 of *TRPM1* present in 13 Ashkenazi Jewish families. Most patients were myopic (with mean BCVA of 0.40 LogMAR) and all had absent rod responses in full field electroretinography. To the best of our knowledge, this is the largest report of a clinical and genetic analysis of patients affected with CSNB due to *TRPM1* mutations.

## Introduction

Congenital stationary night blindness (CSNB - OMIM #613216) is a clinically and genetically heterogeneous retinal disease which is generally considered as non- progressive or a minimally progressive disease^1^. Similar to other hereditary retinal disorders, CSNB is heterogeneous, displaying all modes of inheritance. The clinical presentation varies and is usually classified into three main types, based on the pattern in electroretinogram (ERG) responses: Schubert-Bornschein (negative pattern of the dark-adapted rod-cone response in which the a-wave is normal or almost normal whereas the b-wave is absent or nearly absent)^2^, Riggs (small negative and positive waves)^3^ or Nougaret (absent rod a-wave)^4^. The Schubert-Bornschein type has further been divided into complete (CSNB1) and incomplete (CSNB2), depending on the complete absence or still partially apparent pure rod response under scotopic conditions. Six genes are currently known to cause autosomal recessive CSNB (arCSNB), five of which, *CABP4*^5,6^, *GRM6*^7^, *SLC24A1*^8^, *GPR179*^9^ and *LRIT3*^10^ account for the disease in a relatively small number of families. *CABP4* is the only gene known to date to cause arCSNB2^11^. Mutations in the sixth gene, *TRPM1*, were reported to be a major cause of arCSNB in patients of different origins^12–15^.

*TRPM1* (transient receptor potential type M group 1) encodes an ion-conducting plasma membrane channel^16^ which is part of the transient receptor potential (TRP) family of channels, first described in Drosophila with phototransduction defects^17,18^. TRP channels are expressed in diverse tissues and cell types facilitating cations current across the membrane^19^. TRP channels respond to numerous stimuli (mechanical, chemical, pH and even light), as well as intra and extra cellular ligands. Mutations in TRP genes have been shown to cause diseases with cardiovascular, neurological, metabolic or neoplastic phenotypes. In the retina, TRPM1 is expressed in the ON-bipolar cells and is required for the depolarizing light response^20^. *TRPM1* mutations were reported to cause arCSNB in a horse breed^21^; followed by three back-to-back publications reporting a large set of causative mutations in human patients using homozygosity mapping^13^ or the candidate gene approach^12,14^. Since then, *TPRM1* mutations were reported worldwide to be a major cause of arCSNB^11,15,21,22^.

In the current study, we recruited Israeli and Palestinian patients with arCSNB and applied homozygosity mapping as a primary tool for gene identification^23^. The analysis revealed a relatively large common region of chromosome 15, harboring *TRPM1*. A subsequent mutation analysis of *TRPM1* revealed four mutations, two of which are founder mutations. The phenotype of *TRPM1*-mutated patients is within the reported clinical spectrum. To the best of our knowledge, this is the largest cohort of *TRPM1*-CSNB patients to be reported in the literature thus far.

## Materials and Methods

### Patient recruitment

The tenets of the Declaration of Helsinki were followed and informed consent was obtained from all patients who participated in this study prior to donation of a blood sample. The study was approved by the ethics committee at St John eye hospital and the institutional review board at Hadassah Hebrew university medical center. All experiments were performed in accordance with relevant guidelines and regulations. Inclusion criteria for participation in this study were the following: a clear diagnosis of CSNB, and a signed informed consent. Peripheral blood samples were obtained from the index patient, other affected and unaffected family members for DNA analysis. Genomic DNA was extracted from blood using the FlexiGene DNA kit (QIAGEN).

### Clinical evaluation

A full ophthalmologic examination was performed on all patients when available, as follows: best-corrected visual acuity (BCVA), refractive error, clinical ocular exam by slit lamp biomicroscopy, full-field electroretinography (ffERG), Goldmann visual fields, ocular coherence tomography (OCT), color, infrared and short-wave fundus autofluorescence (SWAF) imaging. BCVA was measured at each visit of the patient in the clinic and the average of both eyes was taken.

ffERG responses were recorded according to the ISCEV standards using corneal electrodes and a computerized system (UTAS 3000, LKC, MD). In the dark-adapted state, a rod response to a dim blue flash and a mixed cone-rod response to a white flash were acquired. Cone responses to 30-Hz flashes of white light were acquired under a background light of 21 cd/m^2^. All responses were filtered at 0.3–500 Hz and signal averaging was used.

### Single nucleotide polymorphism (SNP) array analysis

Whole genome SNP analysis was performed using 10K/250K Affymetrix system and 6K Illumina (human genome build 37/hg19). A region of homozygosity was determined by a minimal length of 5 cM.

### Mutation analysis

Primers were designed using PRIMER3 software (available in the public domain at http://bioinfo.ut.ee/primer3-0.4.0/, Supplementary Table 1). PCR was performed in 30 µl reaction with 35 cycles according to classical protocols. Mutation analysis was performed by Sanger sequencing of PCR products.

## Results

### Prevalence and genetics of CSNB in Israeli and Palestinian patients

Aiming to estimate the prevalence of CSNB in the vicinity of Jerusalem, we created a database that contains clinical information from the only two centers that perform ERG in the vicinity of Jerusalem: Hadassah-Hebrew University Medical Center and St. John Eye Hospital. Data were collected and verified for patients who reside in the vicinity of Jerusalem only, to reliably assess disease prevalence. Our database contains a total of 161 CSNB patients representing the minimal number of CSNB patients in the vicinity of Jerusalem with an estimated population size of 1,000,000 individuals (based on the Israeli, https://www.cbs.gov.il/EN/pages/default.aspx, and Palestinian, https://www.pcbs.gov.ps/default.aspx, Bureau of Statistics). Hence, the expected prevalence of CSNB in the vicinity of Jerusalem is estimated to be at least 1:6210 individuals. Disease prevalence was found to be higher among Palestinians (1:4090) compared to the Jewish population (1:8155).

We recruited 76 families with CSNB for this study, with the following inheritance patterns: AR in 28 families (37%), consanguineous isolate cases (indicating AR inheritance) in 11 families (14%), X-linked (XL) in 8 families (11%), AD in 3 families (4%); and 26 isolated cases with no consanguinity (34%). The majority of families with AR inheritance pattern were consanguineous (17/28, 61%) and in the remaining AR families the parents share the same ethnic origin, leading to the assumption that most patients are likely to be homozygous for the disease-causing mutation due to identity-by-descent of the corresponding locus.

**Figure 1:**
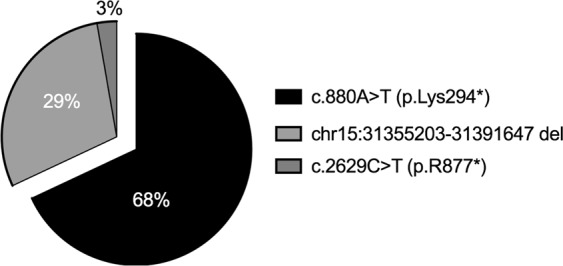
Frequency of *TRPM1* mutations in CSNB patients included in our study. Seventy-four percent of the patients harbor the c.880A>T mutation in a homozygous state.

**Figure 2:**
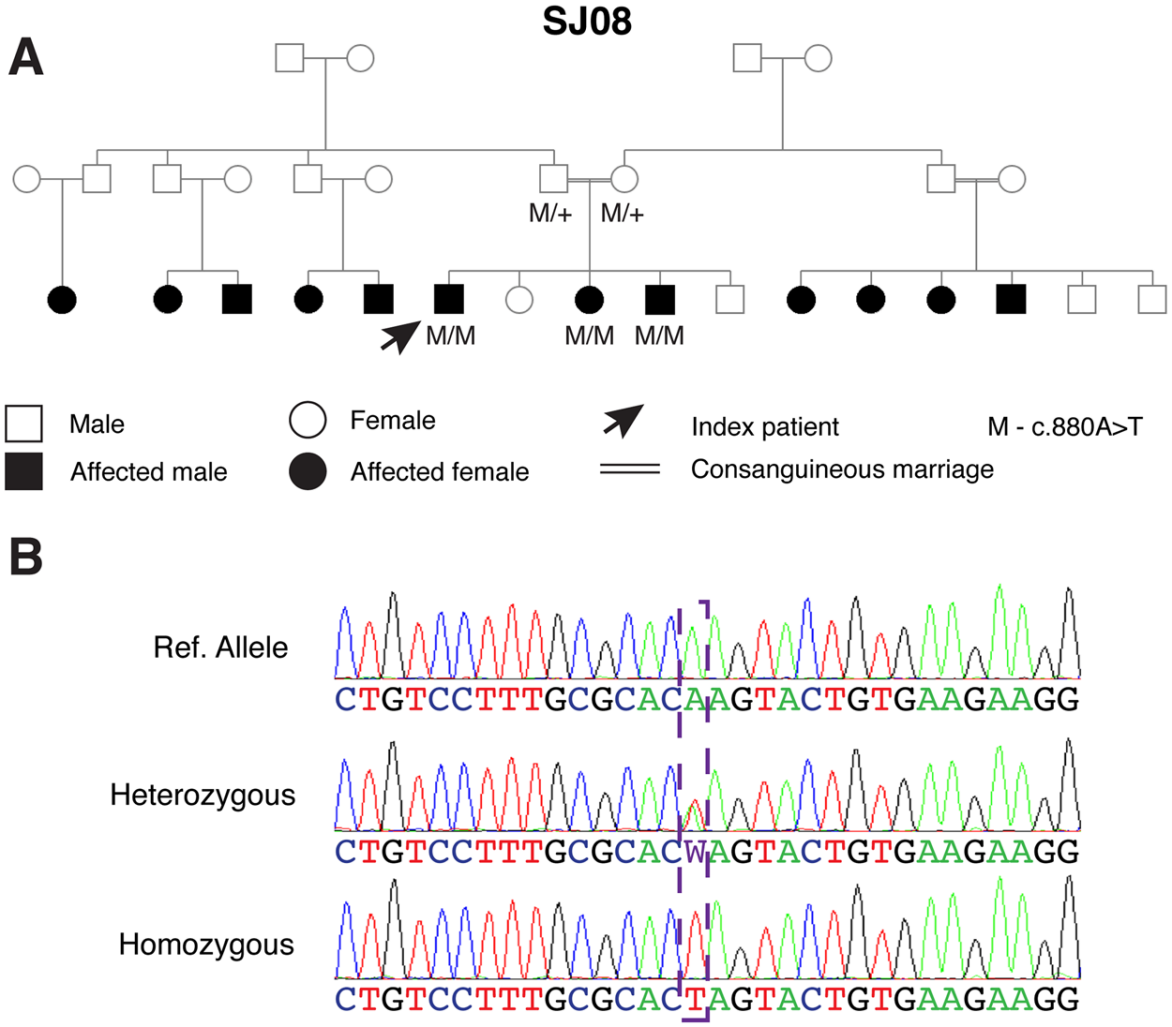
The pedigree (**A**) and chromatogram (**B**) of a family with 12 affected subjects who harbor a homozygous mutation c.880A>T in the *TRPM1* gene.

Due to high frequency of AR or suspected AR cases we decided to focus on identifying the underlying causative genes in these families. We therefore performed homozygosity mapping in eight index patients of Palestinian families and identified a single shared homozygous locus on chromosome 15 in six of the patients (Supplementary Table 2), the two remaining patients were not homozygous for this locus. The shared locus included 30 protein coding genes, one of which is *TRPM1*. Sequencing analysis of *TRPM1* in the six index patients, and subsequent screening of additional patients affected by arCSNB, revealed a total of 4 mutations, two of which are founder (Table 1 and Fig. 1). Biallelic pathogenic *TRPM1* mutations were identified in 36 families (with a total of 72 patients); including 17 out of the 28 AR families (61%) and 19 of the 37 isolate cases (51%). Two founder mutations, that explain the vast majority of cases (97%, Fig. 1), were identified: a novel transversion, c.880A>T, which is predicted to produce a premature termination codon at the 294^th^ residue of the protein (p.Lys294*) was found in 22 Palestinian families (49 patients) residing in Eastern Jerusalem (Fig. 2), and a large genomic deletion (36,445 bp) encompassing exons 2–7 of *TRPM1* was identified in 13 Ashkenazi Jewish families (including 21 patients). The genomic deletion was reported previously in two patients^1,14^, both are from an Ashkenazi Jewish origin as well (personal communication - Marteen Kamermans and Samuel Jacobson). In addition, we identified a novel homozygous nonsense mutation (p.R877*) in a Palestinian family (MOL0325). In a family of Iranian Jewish decent (MOL0015) we identified a single novel heterozygous nonsense mutation (p.W856*) with no evident mutation on the counter allele, and therefore it is not yet clear whether *TRPM1* is the disease-causing gene in this family. All four mutations are expected to either be subjected to nonsense-mediated RNA degradation (NMD) producing no protein or produce a mutant and nonfunctional protein, and therefore these mutations are likely null. One patient, MOL0388-1, was a compound heterozygous for two *TRPM1* variants (a previously reported missense and a silent change), but since we do not have a concrete evidence for its pathogenicity, we did not include this patient in the *TRPM1*-related clinical analysis.

**Table 1:** *TRPM1* mutations identified in this study.

Nucleotide Change	Predicted Amino Acid Change	Exon	Families	ACMG 2015 mutation classification	Reference	Ethnic Origin	^#^of Families (Patients)
**A. Definite Pathogenic Mutations**							
c.880A>T	p.Lys294*	7	MOL0084, MOL0086, MOL0224, MOL0239, MOL0398, MOL0417, MOL0471, MOL0609, MOL0688, MOL0743, MOL0968, MOL1075, MOL1085, MOL1188, MOL1311, MOL1420, SJ0004, SJ0008, SJ0016, SJ0164, SJ0181, SJ0185	PVS1	^1^	Palestinian	22 (49)
chr15: 31355203- 31391647del (exon2–7del)	NA	2–7	MOL0079, MOL0132, MOL0362, MOL0611, MOL0614, MOL0720, MOL0903, MOL0976, MOL1265, MOL1521, MOL1631, RD206-1, RD356-1	PVS1	^1, 12^	Ashkenazi Jews	13 (21)
c.2629C>T	p.R877*		MOL0325	PVS1	^1^	Palestinian	1 (2)
**B. Possible Pathogenic Mutations**							
c.897C>T	p.G299G	7	MOL0388	PP3	^1^		
c.2783G>A	p.R928Q	21	MOL0388	PP3	^15^		
**C. Monoallelic Families**							
c.2567G>A	p.W856*	20	MOL0015	PVS1	^12^	Iran Jew	1(2)

### Clinical data

**Figure 3:**
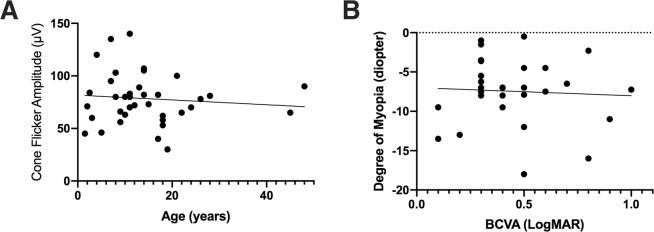
Clinical characteristics of patients with biallelic *TRPM1* mutations. (**A**) Plot of cone flicker (30 Hz) response versus age correlation show very mild decline in older ages. (**B**) Plot of refraction versus BCVA (LogMAR) demonstrates no correlation between visual acuity and degree of myopia. Each point in the graphs represents an average of two eyes.

We clinically evaluated 42 patients with biallelic *TRPM1* mutations. Most patients were diagnosed at a relatively young age due to congenital night blindness (Supplementary Table 3). While some degree of variability exists, all patients (n = 35) for whom refraction data were available were myopic, often to a high degree from birth. The mean refractive error was −7.50 D spherical equivalent (ranging from −1.00 to −18.0 D) with a tendency to show similar values among family members (for example, 3 affected members of family MOL0609 with a refractive error of −8.00, −6.50, and −6.00D, Supplementary Table 3). Interestingly, visual acuity was variably impaired in all patients. Only four patients (out of the 33 for whom BCVA data was available) had decimal acuities of 0.70 (LogMAR of ~0.20) or above, with a mean BCVA of 0.42 (LogMAR of ~0.40, range between 0.10 to 1.0 decimal and LogMAR) for the group. However, our clinical analysis shows that there is no correlation between amplitude of the cone flicker response and age or visual acuity and degree of myopia (Fig. 3).

As is the case in the majority of CSNB patients, fundoscopic findings (when present) were largely minimal (e.g. tilted optic discs, peripapillary atrophy and mild RPE changes), all of which probably reflect the underlying myopia, as can be seen in OCT and SWAF images (Supplementary Fig. 1). For the most part, the fundus exam was within normal limits absent of any changes usually associated with retinal degeneration (such as waxy disc pallor, narrowing of vessels or bone spicule-like pigmentation).

Electroretinographic responses in all patients were characterized by absence of the dark-adapted rod-derived b-wave and a negative pattern of the dark-adapted mixed rod-cone responses, in which the a-waves (reflecting photoreceptor function) were largely preserved while the b-waves (mainly reflecting bipolar cell function) were markedly reduced, which is compatible with the Schubert-Bornschein type of night blindness. Light-adapted cone responses were within normal limits or slightly below lower limit of normal in all but three patients (MOL0611-1, SJ0016-1 and RD356-1).

Our cohort included mainly two groups of patients who are homozygous for a *TRPM1* mutation for whom clinical data was available for at least one parameter: 29 patients were homozygous for c.880A>T and 11 patients for chr15: 31355203-31391647del. Most clinical parameters did not show a significant difference between the two major groups, however, a single parameter (mixed a-wave response) was lower in the chr15: 31355203-31391647del group (139.2 ± 54.2 μV versus 200.9 ± 96.4 μV, p = 0.035 for the c.880A>T group).

## Discussion

The prevalence of CSNB has not been reported yet in many populations, and in only a single study the prevalence was estimated to be “considerably higher than 17 out of 5 million” (or about 1 in 294,000)^24^. As part of the current study, we were able to identify 161 cases with CSNB residing in the vicinity of Jerusalem, reaching a minimal prevalence of 1:6210 individuals. This relatively high prevalence is mainly due to the high rates of consanguineous marriages among Palestinian population (according to the Palestinian central Bureau of statistics the consanguinity rates among Palestinians are estimated to be 45%) and intra-community marriages in the Ashkenazi Jewish population^25^. In the current study, we identified two founder mutations in the *TRPM1* gene: a nonsense mutation that is common in the Palestinian population leading to a disease prevalence of about 1:4090 in this sub-population and a genomic deletion that is common in the Ashkenazi Jewish population leading to a disease prevalence of 1:8155 among all Jews in the vicinity of Jerusalem. In a prevalence analysis of non-syndromic RP in the same region, we previously reported that it is 2.5–3 times higher compared to other reports (1:2086 in the vicinity of Jerusalem compared to an average of 1:5260 in European and American populations)^26^.

Thus far, patients with CSNB due to *TRPM1* mutations have been reported in a number of publications (Supplementary Table 4), and most were summarized by Zeitz *et al*.^1^. This study included 32 index cases who belong to different populations. Here we report an additional set of 36 families (72 patients) which is, as far as we know, the largest set of *TRPM1* cases reported thus far. Interestingly, in this large set of patients mainly two founder mutations are responsible for 97% of solved cases. Of note, *TRPM1* is the most common CSNB gene in the studied populations and is responsible for the disease in 47% of families.

This large cohort of patients who are also clinically characterized, allowed us to study possible correlation between clinical parameters and to identify clinical features that are common or unique to *TRPM1* patients. Our findings are largely similar and further support those reported previously, including the fact that *TRPM1* mutations exclusively cause the complete form of CSNB, there is a high prevalence of moderate to high myopia, and the visual acuity is often suboptimal, suggesting possible dysfunction of cone-derived and not only rod photoreceptor-derived vision^1,27,28^. Interestingly, the phenotypic variability among *TRPM1* patients seems to be lower than that observed in association with other genes causing hereditary retinal disease, including many RP-causing genes, such as *ABCA4* (causing variable phenotypes including Stargardt, cone-rod degeneration, and retinitis pigmentosa) as well as *CACNA1F* which may cause CSNB but also rod-cone and cone-rod degeneration phenotypes^29–31^.

The sub-optimal visual acuity found in the majority of *TRPM1* patients may suggest either macular involvement (which is not evident by fundoscopy and OCT imaging) or could be as a result of the myopia with or without an associated component of amblyopia (dependent upon the degree of refractive error and the age at which the refractive error was corrected). However, our clinical analysis shows that there is no correlation between visual acuity and degree of myopia or the amplitude of the cone flicker response.

CSNB is considered to be a relatively mild retinal phenotype, mainly because it affects night vision (which is less critical for quality of life and function in the modern well-lit world), does not cause retinal degeneration, and there is no or only mild progression with age. However, recent studies, including the data we present here, show that many *TRPM1* patients have impaired visual acuity.

In summary, we report here of the largest cohort of CSNB patients who are genetically diagnosed with *TRPM1* mutations. This cohort can serve as a test population for therapeutic modalities for *TRPM1*. It should be noted, however, that classical gene augmentation therapy using adeno-associated virus (AAV) might be complicated for *TRPM1*, due to the large mRNA transcript encoded by the gene. On the other hand, most of the patients identified here are homozygous for the same nonsense mutation, p.K294*, and might benefit from additional approaches, such as translational read-through-inducing drugs (TRID) that have recently been approved for treating cystic fibrosis. In view of the fact that day-time vision is affected in this subpopulation of CSNB patients, which potentially exposes them to co-morbidity factor associated with vision defects, makes this type of CSNB an attractive target for therapeutic intervention.

## Supplementary information

Supplementary Information

## Data Availability

All data generated or analysed during this study are included in this published article (and its Supplementary Information files).
