# Supplementary material for: TRPM1 Mutations are the Most Common Cause of Autosomal Recessive Congenital Stationary Night Blindness (CSNB) in the Palestinian and Israeli Populations
Source: Sci Rep. 2019 Aug 19;9:12047. doi: 10.1038/s41598-019-46811-7 (PMC6700182; doi:10.1038/s41598-019-46811-7)

TRPM1 Mutations are the Most Common Cause of Autosomal Recessive Congenital Stationary Night Blindness (CSNB) in the Palestinian and Israeli Populations

Alaa AlTalbishi (1,2)*, Lina Zelinger (1,3)*, Christina Zeitz (4), Karen Hendler (1), Prasanthi Namburi (1), Isabelle Audo (4,5,6), Claudia Yahalom (1), Samer Khateb (1,4), Eyal Banin (1)* and Dror Sharon (1)*

(1) Department of Ophthalmology, Hadassah-Hebrew University Medical Center, Jerusalem 91120, Israel

(2) St. John of Jerusalem Eye Hospital Group, Jerusalem 91198

(3) Current affiliation: Neurobiology Neurodegeneration & Repair Laboratory, National Eye Institute, NIH

(4) Sorbonne Université, INSERM, CNRS, Institut de la Vision, Paris, France.

(5) CHNO des Quinze-Vingts, DHU Sight Restore, INSERM-DGOS CIC 1423, Paris, France

(6) Institute of Ophthalmology, University College of London, London, UK

* <u>Equally contributed</u>

Supplementary Table 1: Sequence of *TRPM1* primers used in the current study.

Exon #	Primer Forward	Primer Reverse
2	5'-AGAATGGACTCTGATGATGG-3'	5-'GCCACCCTTCTGGACTC-3'
7	5'-ACATGCCTGAATTCCTTC-3'	5-'TTAAACACGTTTGGAATGTC-3'
20	5'-ATCTAACTTCCCAGTTGTGG-3'	5-'TTTTGTTTTCGAGATGGAC-3'
26	5'-TAGGAGTGGCGGAGAAG-3'	5-'TAAAACAGGAGAACTCGG-3'

Supplementary Table 2: The homozygous region harboring *TRPM1* identified in arCSNB patients.

**Patient number**	193-1	796-1	86-1	224-1	471-1	743-1	398-1	417-1
**Size in Mbp of Homozygous region**	9.23	18.82	9.90	9.38	32.11	7.64	9.54	10.88
**Shared Locus size**	8.22		3.59		5.97		8.35	
***TRPM1* mutation**	None		c.880A>T (p.Lys294*)					
**Shared Locus interval**			30,151,789-33,414,466 (3.26 Mb)					

Supplementary Table 3: Clinical data of patients with *TRPM1* mutations

Patient ID	Origin	Consanguinity	Mutation	Age (sex)	Refractive error	VA	Full-Field ERG				
							Flicker		Mixed amplitude		Rods
							Amp	IT	a-wave	b-wave	
MOL0084-1	Palestinian	No	c.880A>T	26 (M)	High myopia	NA	78	31	270	139	Absent
MOL0084-2	Palestinian	No	c.880A>T	7 (M)	NA	0.1	135	32.3	300	142	Absent
MOL0086-1	Palestinian	Yes 2:2	c.880A>T	14 (F)	NA	NA	105	32.2	339	214	Absent
MOL0224-1	Palestinian	Yes	c.880A>T	11 (M)	-6.8	0.3	80	32	281	193	Absent
MOL0239-1	Palestinian	Yes 2:2	c.880A>T	8 (F)	NA	NA	103	32	304	189	Absent
MOL0239-2	Palestinian	Yes 2:2	c.880A>T	7 (F)	NA	NA	95	32.4	235	63	Absent

MOL0398-1	Palestinian	No	c.880A>T	14 (F)	-12.0	0.5	107	32.4	265	163	Absent
MOL0398-3	Palestinian	No	c.880A>T	22 (F)	-9.50	0.1	65	34	66	60	Absent
MOL0417-1	Palestinian	No	c.880A>T	10 (F)	-9.50	0.4	80	35.4	Decreased	Decreased	NA
MOL0417-4	Palestinian	No	c.880A>T	15 (M)	High Myopia	0.7	73	33.2	180	120	Absent
MOL0471-1	Palestinian	Yes 2:2	c.880A>T	18 (M)	High Myopia	NA	62	36.3	174	145	Absent
MOL0609-1	Palestinian	Yes 2:2	c.880A>T	11 (F)	-8.00	0.4	140	32.0	NA	NA	NA
MOL0609-2	Palestinian	Yes 2:2	c.880A>T	11 (M)	-6.50	0.7	70	31.5	NA	NA	Absent
MOL0609-4	Palestinian	Yes 3:3	c.880A>T	4 (M)	-6.00	NA	120	34	WNL	Severely Reduced	NA
MOL0688-1	Palestinian	No	c.880A>T	48	NA	0.3	90	30.8	198	149	Absent

				(F)							
MOL0743-1	Palestinian	Yes 2:2	c.880A>T	13 (F)	-7.00	0.5	89	32.0	261	115	Absent
MOL968-1	Palestinian	Yes 2:2	c.880A>T	21 (M)	-13.00	0.2	100	32.5	177	89	Absent
MOL1075-2	Palestinian	Yes 2:2	c.880A>T	8 (M)	-4.50	0.6	103	32.5	WNL	Severely Reduced	NA
MOL1085-1*	Palestinian	Yes 2:3	c.880A>T	8 (F)	-6.25	0.3	80	33.5			Severely reduced
MOL1188-1*	Palestinian	Yes 2:2	c.880A>T	9 (F)	-7.50	0.3	66	36.5			Absent
MOL1311-1*	Palestinian	No	c.880A>T	2 (M)	-8.00	0.3	71	30.5	WNL	Severely Reduced	NA
MOL1420-1	Palestinian	Yes 3:2	c.880A>T	9 (M)	-4.50	0.5	82	32			
SJ4-1	Palestinian	Yes 2:2	c.880A>T	3 (F)	-1.0	0.3	60	37	110	68	Absent

SJ8-1	Palestinian	Yes 2:2	c.880A>T	13 (M)	-5.50	0.3					39
SJ16-1	Palestinian	Yes 2:2	c.880A>T	19 (M)	-3.50	0.3	30	31	30	56	Absent
SJ164-1	Palestinian	Yes 2:2	c.880A>T	14 (F)	-7.0	0.4	82	34	40	73	43
SJ164-2	Palestinian	Yes 2:2	c.880A>T	8 (M)	-7.50	0.6					NA
SJ181-1	Palestinian	Yes 2:2	c.880A>T	5 (M)	-7.25	1.0	46	30	38	47	Absent
SJ185-1	Palestinian	Yes 2:2	c.880A>T	18 (M)	-5.50	0.5					NA
MOL0325-1	Palestinian	Yes 2:2	c.2629C>T	10 (M)	-13.50	0.1	63	33.8	148	77	Absent
MOL0325-3	Palestinian	Yes 2:2	c.2629C>T	28 (F)	-18.0	0.5	81	32	121	75	Absent
MOL0079-1	Ashkenazi	No	chr15: 31355203-	24	-7.90	0.5	70	30.3	175	82	Absent

	Jewish		31391647del	(M)							
MOL0132-1	Ashkenazi Jewish	No	chr15: 31355203-31391647del	9 (F)	-7.50	NA	56	34.5	98	110	Absent
MOL0362-1	Ashkenazi Jewish	No	chr15: 31355203-31391647del	12 (M)	-2.30	0.8	72	30.8	142	92	Absent
MOL0611-1	Ashkenazi Jewish	No	chr15: 31355203-31391647del	17 (M)	-11.0	0.9	40	34.3	91	65	Absent
MOL0614-1	Ashkenazi Jewish	No	chr15: 31355203-31391647del	17 (F)	-1.50	0.3	82	31.8	196	131	Absent
MOL0720-1	Ashkenazi Jewish	No	chr15: 31355203-31391647del	45 (M)	Myopia	0.3	65	32.5	149	89	Absent
MOL0903-1	Ashkenazi Jewish	No	chr15: 31355203-31391647del	18 (M)	-7.00	0.4	53	34.0	86	78	Absent
MOL0976-1	Ashkenazi Jewish	No	chr15: 31355203-31391647del	11 (M)	-3.60	0.3	83	32.5	102	49	Absent
MOL1613-1	Ashkenazi Jewish	No	chr15: 31355203-31391647del	36 (M)	-16	0.8	58	31.5	103	76	Absent

RD206-1	Ashkenazi Jewish	No	chr15: 31355203-31391647del	2.5 (F)	-9.25	FF M	84	35	WNL	Severely Reduced	NA
RD356-1	Ashkenazi Jewish	No?	chr15: 31355203-31391647del	1.5 (F)	-5.0	FF M	45	33			

* Short protocol.

Refractive error, visual acuity and ERG responses were largely symmetrical, so presented as average of the two eyes. Refractive error given as spherical equivalent, in diopters.

Visual acuity presented as decimal

ffERG=Full field electroretinogram including the followings: Rod response b-wave amplitude (in µV, normal > 200µV); Mixed cone-rod a/b wave (in µV, normal a-wave > 90µV, normal b-wave > 400µV); Cone response (in µV, normal > 60µV), implicit time (in msec, normal ≤ 33msec).

F, female; M, male; NA, not available, IT - Implicit Time, CSUM- central, steady, unmaintained, FFM- fix, follow and maintain,

Supplementary Table 4: Previously reported *TRPM1* cases

Year of Report	Number of Familes	Number of Patients with Biallelic Mutations	Reference
2009	3	8	(Li *et al.* 2009)
2009	10	10	(Audo *et al.* 2009)
2009	6	6	(van Genderen *et al.* 2009)
2010	3	3	(Nakamura *et al.* 2010)
2012	1	1	(Audo *et al.* 2012)
2013	9	10	(Bijveld *et al.* 2013)
2014	6	6	(Malaichamy *et al.* 2014)
2015- total thus far- based on	32	32	(Zeitz *et al.* 2015)

review			
Total thus far			
Current report	33	68	

The table includes patients with bi-allelic *TRPM1* mutations.

Supplementary Figure 1: Fundus photos , SWAF and OCT of an example case (SJ 185-1) showing the peripapillary atrophy , fundus tessellation and posterior staphyloma (as shown in the OCT).

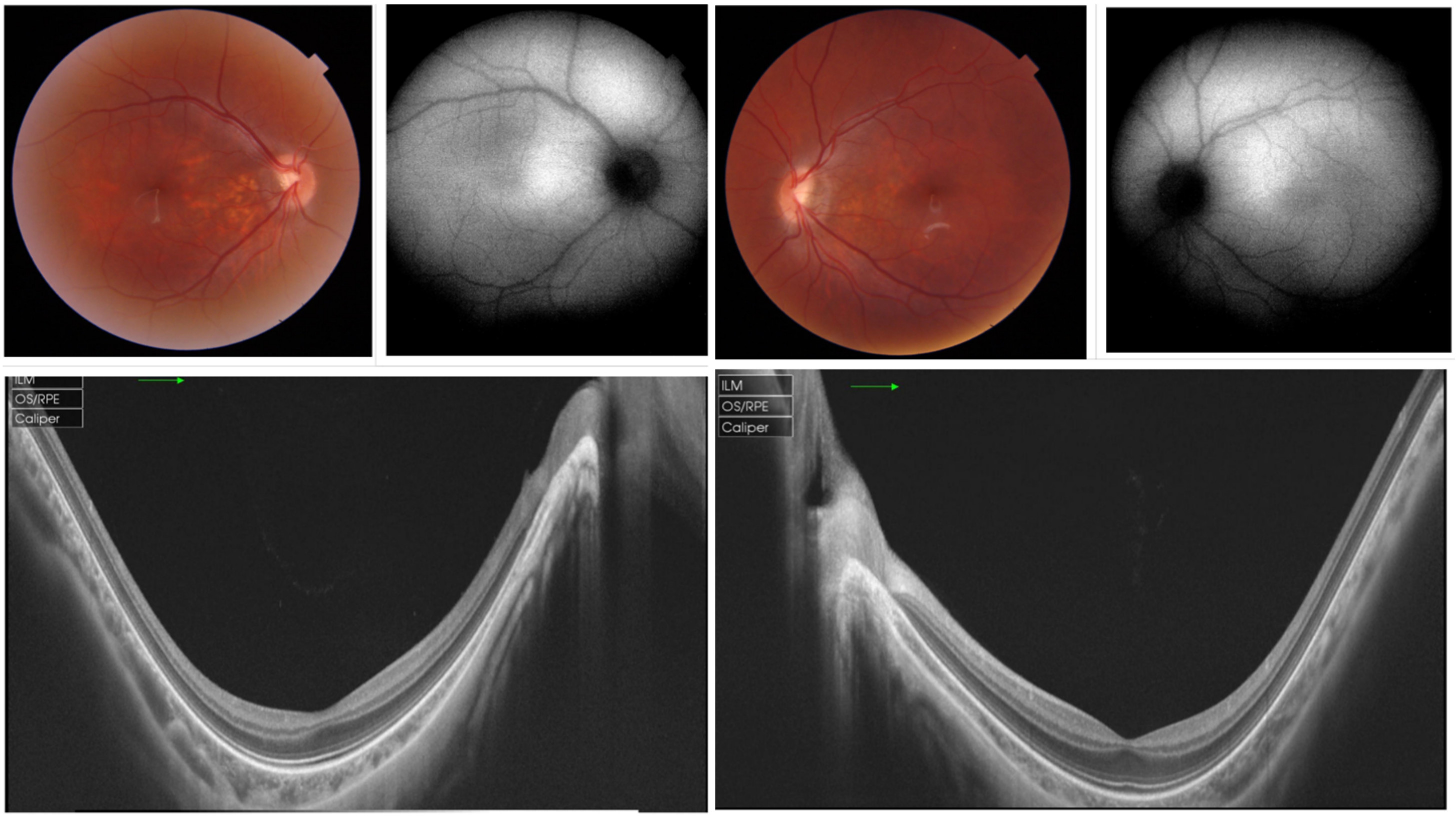

Supplement: Supplementary file 1 — Supplementary Information [file 41598_2019_46811_MOESM1_ESM.pdf]
